# Feasibility of telephone-based telemedicine for gynecologic oncology follow-up in Brazil’s public health system: a descriptive pilot study

**DOI:** 10.1016/j.clinsp.2026.100968

**Published:** 2026-04-25

**Authors:** Raphael Federicci Haddad, Fernanda Mazziero Pires, Luiza Perez, Gustavo Yano Callado, Juliana Lomanto, Flavia Paiva Proenca Lobo Lopes, Eduardo Cordioli, Vanessa Alvarenga-Bezerra, Renato Moretti-Marques

**Affiliations:** a Hospital Israelita Albert Einstein, São Paulo, SP, Brazil; b Hospital Municipal Vila Santa Catarina Dr. Gilson de C. Marques de Carvalho, São Paulo, SP, Brazil; c Faculdade de Medicina, Universidade Federal do Rio de Janeiro, Rio de Janeiro, RJ, Brazil; d New York Presbyterian, Weill Cornell Medicine, New York, NY, USA

**Keywords:** Gynecological cancer, Gynecological surgery, Telemedicine, Patient satisfaction

## Abstract

**Background:**

Telemedicine expanded rapidly during COVID-19 and may reduce logistical burdens for oncology patients. Evidence on patient satisfaction within Brazil’s public health system (SUS) remains limited. Here, the authors evaluated the feasibility of telephone-based telemedicine for gynecologic oncology follow-up.

**Methods:**

The authors conducted a cross-sectional mixed-methods pilot study at a public gynecologic oncology clinic in São Paulo (March 2023 – February 2024). Established patients attending follow-up visits that did not require a physical exam were included; new patients were excluded. Telemedicine visits were conducted by phone. The PSQ-18 was translated and adapted to the SUS context (two cost items removed: 16-items total). Item-level satisfaction is presented for in-person and telemedicine visits. Semi-structured interviews explored experiences and barriers.

**Results:**

From 54 eligible patients, 18 were enrolled (in-person = 10; telemedicine = 8). Item-level PSQ-18 responses indicated high satisfaction in both groups, though direct comparison is limited by baseline differences and limited statistical power. Regarding logistics, the telemedicine group reported low rates of out-of-pocket expenses (1/7, 14.3%) and missed work (0/8), whereas these burdens were frequently reported in the in-person group (77.8% and 33.3%, respectively). Physicians reported full goal achievement (100%) and patient understanding (100%) for telemedicine encounters. Interviews highlighted convenience, reduced travel/time costs, and ease of use; barriers included technology access, digital literacy, and lack of visual cues in audio-only calls.

**Conclusions:**

In this SUS setting, telephone-based telemedicine for appropriately selected follow-up visits was feasible. Implementation should address digital inclusion and clarify indications that require in-person assessment. Larger studies should validate telemedicine-specific satisfaction measures and assess downstream outcomes.

## Highlights

• Telemedicine showed high patient satisfaction in gynecologic oncology care.
• Remote visits reduced travel, costs, and time compared to in-person visits.
• Telephone-based care was feasible despite limitations in physical examination.
• Patients and providers supported telemedicine as a complementary care strategy.
• Educational and digital access disparities may influence telemedicine adoption.

## Introduction

Telemedicine, defined as the remote provision of healthcare via telecommunication technologies, emerged as a crucial tool in recent years.^1^ It is not a specific clinical service but a set of tools enhancing care access, service delivery, and health education.^2^

While telemedicine cannot fully replace in-person visits ‒ especially when physical exams are required ‒ it improves healthcare accessibility, particularly in remote areas.^3^ In Brazil, where healthcare access is uneven, telemedicine optimizes resource distribution and expands coverage.^4^ In oncology, it reduces travel-related time and costs.^5^

Patient satisfaction, a key healthcare quality metric, depends on factors like care quality, accessibility, cost, and waiting times.^5^^,^^6^ In the realm of telemedicine, connection stability, platform usability, and patient-provider interaction hold substantial relevance to its feasibility.^7^ In oncology, preoperative and follow-up telemedicine visits enhance convenience and reduce anxiety.^5^^,^^6^

In gynecologic oncology, the need for a pelvic examination often determines visit modality. Encounters focused on test/result review, treatment counseling, and stable symptom follow-up are well-suited to telemedicine, whereas new concerning symptoms, examination-dependent assessments, and sensitive conversations (e.g., breaking bad news) generally warrant in-person visits. Accordingly, modality selection should be individualized and guided by clinical urgency, the need for physical examination, and patient preference.^8^^,^^9^

International studies report that telemedicine satisfaction levels in gynecologic oncology are comparable to or higher than in-person visits.^10^^,^^11^ In Brazil, studies indicate feasibility and high satisfaction rates, particularly among low-income populations.^12^ To our knowledge, no comparative studies in gynecologic oncology have been published within Brazil’s public health system (*Sistema Único de Saúde*, SUS) context. The present study aims to assess the feasibility of telephone-based telemedicine for follow-up visits not requiring pelvic examination within a public gynecologic oncology service. As exploratory outcomes, the authors also assessed patient satisfaction and healthcare providers’ perceptions across in-person and phone-based telemedicine.

## Methods

### Study design

**Fig. 1:**
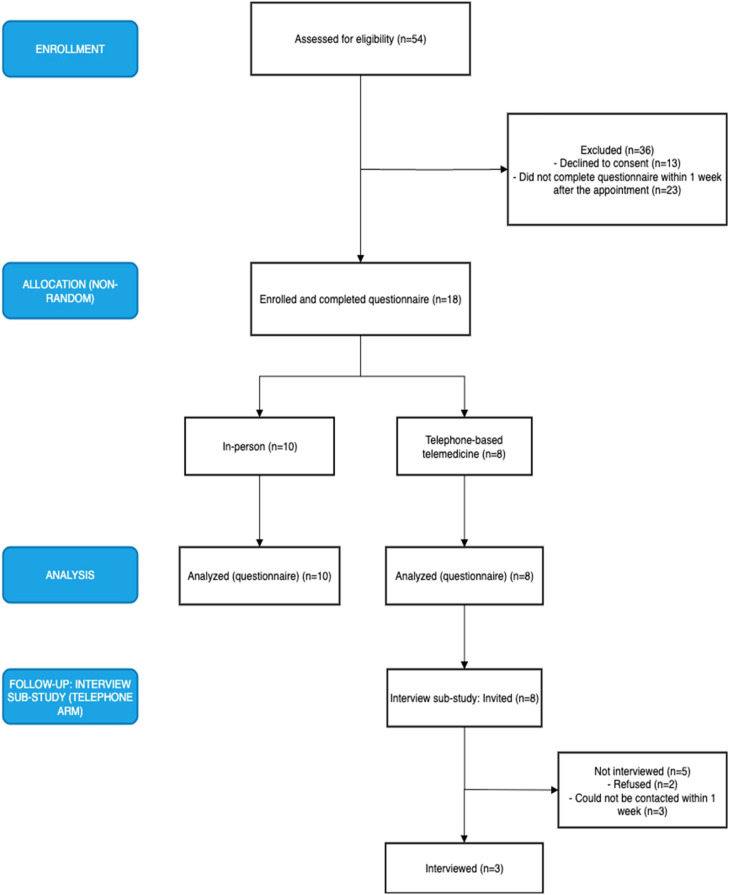
Flow diagram of recruitment, allocation (non-random), follow-up, and analysis.

This was a pilot mixed-methods study. Data collection, from both in-person and telemedicine visits, took place at Hospital Municipal Vila Santa Catarina between March 2023 and February 2024. Telemedicine visits were conducted by phone; video was not routinely used during the study period. The authors report the full recruitment flow and reasons for non-participation in Fig. 1.

All study participants, including patients and healthcare professionals, provided Informed Consent (ICF) electronically. The consent form was automatically sent to participants upon agreeing to partake in the research, and it was completed if they wished to proceed.

This study was approved by the Institutional Review Board (CAAE 57,038,722.9.0000.0071). The study was conducted and reported in accordance with the STROBE Statement guidelines.

### Eligibility criteria

The authors included established gynecologic oncology patients (≥ 18-years) scheduled for follow-up visits that did not require a physical examination. Such visits included follow-ups with ancillary test results, preoperative counseling, pathology report discussions, clinical complaints not requiring in-person assessment, and referral visits to other specialties following a decision made by the Gynecologic Tumor Board. Patients were excluded if they were unable to provide informed consent due to illiteracy or cognitive impairment, had urgent or severe clinical complaints requiring immediate medical attention, or lacked the minimal technological resources necessary for telemedicine visits, such as a phone or computer. Newly diagnosed patients were not included. The recruitment and enrollment process is further detailed in Fig. 1.

### Allocation of visit modality

Allocation of visit modality: Visit modality was non-randomized and determined by shared clinical decision-making. Given that the need for physical examination was an exclusion criterion, allocation focused on the anticipated nature of the consultation and patient preference. Telemedicine was offered when the physician anticipated 'good' or 'neutral' news (e.g., stable surveillance, normal test results) and the patient consented to the remote format. Conversely, in-person visits were maintained for sensitive discussions (e.g., suspicion of recurrence) or if the patient preferred face-to-face contact. This pragmatic allocation reflects real-world practice and introduces selection bias, which the authors acknowledge in the discussion.

### Setting and health system context

Care was delivered within Brazil’s publicly funded Sistema Único de Saúde (SUS), where visits and treatments are provided at no direct cost to patients. This context informed instrument adaptation (removal of cost-sharing items) and the focus on access and logistics.

### Data collection

Eligible gynecologic oncology patients were invited to participate and provided informed consent either in person or electronically through a secure link (e-mail, WhatsApp, or SMS), powered by REDCap.^13^ Participants completed the questionnaire using their own devices. The electronic form began with a PDF of the consent form and required explicit agreement to proceed. Demographic and clinical data, as well as the classification of visit purpose (e.g., good news, uncertain, or palliative), were entered by healthcare professionals and extracted from electronic medical records. Patients answered the Patient Satisfaction Questionnaire-18 (PSQ-18) satisfaction scale, questions about telemedicine expectations, and items related to direct and indirect visit costs (e.g., travel, meals, time). Phone/internet costs were excluded. At the end of each visit, physicians recorded whether visit goals were achieved and if patients understood the information provided, based on the patients’ verbal summary.

### The patient satisfaction questionnaire-18

The authors selected the PSQ-18 as a concise, previously validated measure of patient satisfaction.^14^ As no Brazilian Portuguese version was available, the authors conducted a structured translation and cultural adaptation: two independent forward translations (one informed, one naïve), reconciliation by the translators with the study coordinator, and two independent back-translations by native English speakers not involved in earlier steps.

Discrepancies were resolved by consensus, preserving conceptual equivalence with the original instrument. To ensure the instrument accurately reflected the reality of Brazil’s universal healthcare system (SUS), the authors removed two finance-related items (original items 5 and 7) to maintain content relevance for the study population. Consequently, the 16-item inventory was used to generate descriptive insights into patient perception, with analysis focused on individual item performance to map the specific experience of the telemedicine workflow. Consequently, scores are presented as exploratory descriptive data rather than validated measures of satisfaction, and no composite scores were calculated. The full forward/back-translation matrix appears in Supplemental Table 1.

For analysis, the authors retained the original positive/negative wording conventions and reported item-level results rather than composite scores. The questionnaire was administered electronically, and the authors prespecified a 1-week (≤7-days) post-visit completion window to minimize recall bias. As this was a pilot study, formal validation was not conducted and could be addressed in future work.

### Interviews

The authors invited all participants of the telemedicine group to engage in an interview to further explore their perceptions and experiences with telemedicine. Additionally, the healthcare professionals caring for gynecologic oncology patients within the same public health system were invited to participate in one-on-one interviews as well. The aim was to explore their insights and experiences regarding the use of telemedicine in gynecologic oncology. Purposive sampling was used: all patients in the telemedicine group were invited, and to ensure a broad range of perspectives, one professional with over 10-years of medical experience (advanced practice provider) and two with <10-years of experience (gynecology oncology trainees) within the same clinical team were intentionally selected. The selection of professional participants was designed to elicit a broad range of professional perspectives rather than to ensure statistical generalizability. Only three patients of the telemedicine group agreed to participate, while three declined or could not be scheduled due to time constraints or lack of interest, and the remaining participants could not be reached. Similarly, three clinicians participated, and one invited clinician could not be reached.

Interviews lasted approximately 10-minutes and followed structured guides with easily interpretable questions. All interviews were transcribed verbatim, and identifiable information was excluded. Data were analyzed to identify common narrative elements. Given the pilot nature and limited sample size, the analysis focused on descriptive patterns to provide anecdotal context to the quantitative findings, rather than achieving theoretical saturation. Separate interview guides were developed for patients and healthcare professionals, accounting for the unique perspectives of each group. Separate semi-structured guides were developed for patients and clinicians, adapted from instruments previously published by Adake et al.,^15^ pilot-tested for clarity, and provided in Supplemental Table 2. The guides contained 12 questions covering perspectives and experiences, accessibility, barriers, efficacy, and cost. Qualitative reporting followed the Consolidated criteria for Reporting Qualitative Research (COREQ), which is a 32-item checklist for interviews and focus groups (Supplemental Table 3).

### Statistical analysis

Statistical analyses were performed with IBM SPSS Statistics v29.0 (IBM Corp.) and R v4.3.2 (R Foundation). Categorical variables are presented as absolute and relative frequencies, whereas continuous variables are described by median, Inter-Quartile Range (IQR), and observed minimum‒maximum values. The use of medians was warranted by the pronounced asymmetry of all continuous distributions, verified by inspection of boxplots and by the Shapiro-Wilk normality test.

Group differences between in-person and telephone-based telemedicine visits were assessed item-by-item for the adapted 16-item PSQ-18. Due to the pilot nature of this study and baseline imbalances, no hypothesis tests were performed. Missing data were managed via available-case analysis without imputation. Item-level denominators (n/N) are explicitly reported in the tables and figures.

## Results

### Demographic and clinical characteristics

Among the 54 eligible women, 18 were included in the study. Of these, ten attended in-person visits (55.6%), while eight were seen via telemedicine (44.4%). The groups differed substantially at baseline, including cancer type, time since diagnosis, education level, race, and provider seniority. Due to incomplete data for certain demographic variables, such as ethnicity (n = 16), denominators vary across Table 1 to reflect the number of available responses. The percentages are calculated based on the number of available responses for each specific item. Table 1, Table 2, Table 3, Table 4 summarize demographics, clinical characteristics, visit logistics, and clinician-reported assessments.

**Table 1:** Demographic data.

	Total (18^a^)	In-person (10^a^)	Telephone (8^a^)
Age (years)	62.5 [49.3; 69.5]	54 [48; 68]	66 [56; 72]
Ethnicity			
White	9/16 (52.9)	5/9 (55.6)	4/7 (57.1)
Black	1/16 (5.8)	1/9 (11.1)	0/7 (0.0)
Mixed race	6/16 (35.2)	3/9 (33.3)	3/7 (42.9)
Marital status			
Single	7/16 (43.7)	4/9 (44.4)	3/7 (42.9)
Married	5/16 (31.2)	4/9 (44.4)	1/7 (14.3)
Divorced	3/16 (18.7)	0/9 (0.0)	3/7 (42.9)
Widowed	1/16 (6.2)	1/9 (11.1)	0/7 (0.0)
Education level			
Elementary school	7/16 (43.75)	6/9 (66.7)	1/7 (14.3)
High school	7/16 (43.75)	2/9 (22.2)	5/7 (71.4)
Higher education	2/16 (12.5)	1/9 (11.1)	1/7 (14.3)
Household income			
Up to 2 minimum wages	9/16 (56.2)	6/9 (66.7)	3/7 (42.9)
From 2 to 4 minimum wages	5/16 (31.3)	2/9 (22.2)	3/7 (42.9)
From 4 to 10 minimum wages	2/16 (12.5)	1/9 (11.1)	1/7 (14.3)
Has internet access? (yes)	16/17 (94.1)	8/9 (88.9)	8/8 (100.0)
Is the internet used easy to use and/or easily accessible? (yes)	14/16 (87.5)	7/8 (87.5)	7/8 (87.5)

Qualitative data are presented as n/total (%); Quantitative data are presented as median [1st; 3rd quartile]; Denominators vary (n = 16 to 18) due to missing data for certain items; values shown as n/N (%).

a Missing data due to a physician's lack of input in the REDCap platform.

**Table 2:** Patients’ clinical data grouped by type of visit.

	Total (18^a^)	In-person (10^a^)	Telephone-based telemedicine (8^a^)
Current cancer type^b^			
Cervical	5/17 (29.4)	1/9 (11.1)	4/8 (50.0)
Ovarian	2/17 (11.8)	1/9 (11.1)	1/8 (12.5)
Endometrial	7/17 (41.2)	5/9 (55.6)	2/8 (25.0)
Other	3/17 (17.6)	2/9 (22.2)	1/8 (12.5)
Months since current cancer diagnosis	10 [4; 24]	6 [4; 24]	15 [7; 36]
Smoking (yes)	2/16 (12.5)	0/9 (0.0)	2/7 (28.6)
Alcohol consumption (yes)	1/16 (6.2)	1/9 (11.1)	0/7 (0.0)
Menopause (yes)	14/17 (82.3)	7/9 (77.8)	7/8 (87.5)
Age at menopause in years (years)	46 [44; 52]	46 [44; 52]	45 [38; 53]
Years since menopause	15 [8; 21]	13 [2; 20]	18 [15; 29]
Pain visual analog scale from 0 to 10^c^	0 [0; 0]	0 [0; 0]	0 [0; 0]
Comorbidities	7/17 (41.1)	4/9 (44.4)	3/8 (37.5)
Diabetes	3/7 (42.8)	2/4 (50.0)	1/3 (33.3)
Hypertension	3/6 (50)	2/3 (66.7)	1/3 (33.3)
Obesity	1/6 (16.6)	1/3 (33.3)	0/3 (0.0)
Other comorbidity	5/7 (71.4)	3/4 (75.0)	2/3 (66.7)

Qualitative data are presented as n/total (%); Quantitative data are presented as median [1st; 3rd quartile]; Denominators vary due to item non-response; values shown as n/N (%).

a Missing data due to physician lack of input in the REDCap platform.

b Cancer type reflects current primary diagnosis.

c 0 refers to no pain and 10 is the worst pain.

**Table 3:** Visit characteristics and logistics.

	In-person (10^a^)	Telephone-based telemedicine (8^a^)
Visit primarily conducted by		
Attending Physician / Supervisor	2/9 (22.2)	0/7 (0.0)
First-year Gynecologic Oncology Fellow	2/9 (22.2)	6/7 (85.7)
Gynecologic Endoscopy Fellow	1/9 (11.1)	0/7 (0.0)
OB/GYN Resident	3/9 (33.3)	0/7 (0.0)
Undergraduate Medical Student	0/9 (0.0)	1/7 (14.3)
Others	1/9 (11.1)	0/7 (0.0)
Total	9/9 (100.0)	7/7 (100.0)
Main reason for the visit (as perceived by the physician)		
Good news, relief, curative diagnosis	4/9 (44.4)	4/8 (50.0)
Apprehensive (e.g., awaiting care and/or surgery)	3/9 (33.3)	4/8 (50.0)
Bad news (e.g., tumor recurrence, surgical complications)	2/9 (22.2)	0/8 (0.0)
Discouraging news (e.g., palliative care, intractable pain/dysfunction)	0/9 (0.0)	0/8 (0.0)
Total	9/9 (100.0)	8/8 (100.0)
Patient's expectation for the visit		
Good news, relief, curative diagnosis	4/6 (66.7)	2/5 (40.0)
Apprehensive (e.g., awaiting care and/or surgery)	1/6 (16.7)	2/5 (40.0)
Bad news (e.g., tumor recurrence, surgical complications)	0/6 (0.0)	0/5 (0.0)
Discouraging news (e.g., palliative care, intractable pain/dysfunction)	0/6 (0.0)	0/5 (0.0)
No expectations	1/6 (16.7)	1/5 (20.0)
Total	6/6 (100.0)	5/5 (100.0)
Whether the patient had any expenses related to the visit (transportation, food, or other)?	7/9 (77.8)	1/7 (14.3)
Whether the patient missed work or school (formal or informal)	3/9 (33.3)	0/8 (0.0)
Whether the patient was with a companion	7/9 (77.8)	2/8 (25.0)

OB/GYN, Obstetrics and Gynecology; Qualitative data are presented as n/total (%); Quantitative data are presented as median [1st; 3rd quartile]; Denominators vary due to item non-response; values shown as n/N (%).

a Missing data due to physician lack of input in the REDCap platform.

**Table 4:** Physician assessments.

	In-person (10^a^)	Telephone-based telemedicine (8^a^)
Physician considers the objective was achieved?		
No	2/8 (25.0)	0/6 (0.0)
Yes	6/8 (75.0)	6/6 (100.0)
Physician considers the patient understood?		
Fully understood	6/8 (75.0)	6/6 (100.0)
Partially understood, without impact on actions	2/8 (25.0)	0/6 (0.0)
Partially understood, with impact on actions	0/8 (0.0)	0/6 (0.0)
No adequate understanding	0/8 (0.0)	0/6 (0.0)

Data are presented: n/total (%); Denominators vary due to item non-response; values shown as n/N (%).

a Missing data due to physician lack of input in the REDCap platform.

Just one patient from those who answered the identification question was identified as black. Notably, there were no patients who self-identified as black in the telemedicine group. Additionally, a higher proportion of telemedicine participants had completed secondary or post-secondary education compared to those in the in-person group.

The most prevalent cancer type was endometrial cancer (41.2%). Among patients who attended in-person visits, endometrial cancer was also the most frequently diagnosed (55.6%). In contrast, cervical cancer was the most common diagnosis among those who had telemedicine visits (50%). Participant clinical characteristics are summarized in Table 2.

### Medical appointments

The majority of visits in both groups were classified by physicians as delivering positive or concerning news (77.8% and 100%, respectively). In the in-person group, 33.3% of participants missed course or work due to the visit, whereas no absences were reported in the telemedicine group. Furthermore, 77.8% of participants in the in-person group incurred additional expenses related to transportation and meals, compared to only 14.3% in the telemedicine group (Table 3).

Most physicians considered that the visit objectives were achieved, with 75.0% agreement in-person visits and 100.0% in telephone-based telemedicine visits. Similarly, physician assessments of patient comprehension showed that 75.0% of patients fully understood the information in-person visits, while all patients were reported to have fully understood the visit details during telemedicine encounters.

### Comparison of PSQ-18 responses between the two groups

**Fig. 2:**
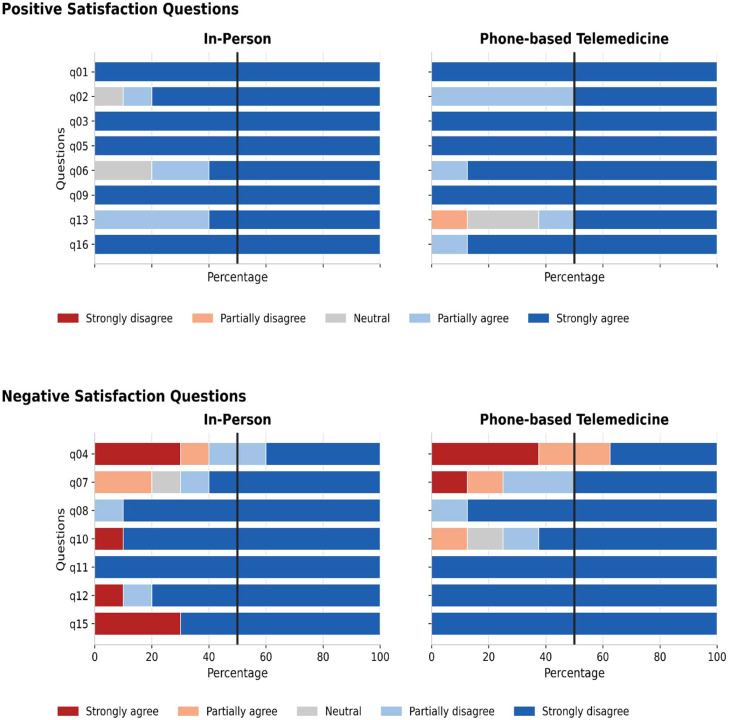
Positive and negative satisfaction question results. Refer to Supplemental Table 1 for detailed descriptions of each question. Negative-question items: higher agreement indicates greater dissatisfaction.

This comparative item-by-item analysis showed that most participants expressed partial or full agreement with positive statements and partial or complete disagreement with negative statements (Supplemental Table 4 and 5). As shown in Fig. 2, there was a higher level of agreement with negative statements in the in-person group compared to the telemedicine group. For instance, in item 15 (a negative statement), 30% of participants in the in-person group agreed, whereas 100% of participants in the telemedicine group disagreed.

The final part of the questionnaire consisted of a free-text question for patients to leave their comments, which was completed by 11 patients (Supplemental Table 6).

### Qualitative interviews with patients

One-on-one interviews with three patients revealed eight themes related to the use of telemedicine in gynecologic oncology care. While these findings are illustrative given the limited sample size (n = 3), participants consistently prioritized convenience, ease of use, and technological assistance. One patient noted: “*[Telemedicine was useful because of the] distance. I live in another state, and it’s too far for me*”.

Telemedicine was valued for reducing travel, saving time, and lowering costs. Patients living far from the hospital appreciated avoiding unnecessary trips for follow-ups or test results, citing savings on transportation and physical strain. Another remarked: *“[With telemedicine] I save time and the stress of traveling. [Travel] involves fuel expenses”*.

Most patients found telephone-based telemedicine visits sufficient, though some preferred video calls for better interaction: *“I would like it to be via video so I can see who I’m talking to and get to know the doctor”*. Overall, patients were satisfied, especially for follow-ups, but recognized that severe or complex cases required in-person visits for physical exams and closer interaction.

Despite its benefits, some noted limitations, such as difficulty in clinical assessments and a lack of nonverbal cues in audio calls. One preferred in-person visits: *“A visit would be better face-to-face”*. Another voiced concerns about identifying telemedicine calls, fearing scams or fraud.

### Healthcare professionals’ perspectives on telemedicine

All professionals reported satisfaction with telemedicine when appropriately indicated. Regarding practicality, they highlighted improved access, particularly for patients with physical limitations: *“It facilitates care for bedridden patients or those with mobility issues”*. However, one professional noted that older patients often struggle with technology, hindering its use.

The choice of visit method was also discussed, with a preference for video calls due to their ability to capture facial expressions and general patient condition. Still, telephone-based telemedicine visits were considered viable and, in many cases, did not compromise care quality. One professional emphasized balancing accessibility and usability to maximize telemedicine’s potential.

A significant limitation, particularly in gynecologic oncology, was the inability to conduct physical exams. One professional stated, *“The challenge I see in oncologic gynecology telemedicine is the physical exam”.* Delivering bad news and providing support during challenging moments were also cited as cases where in-person care is preferable.

Regarding remuneration, most professionals agreed that telemedicine fees should be equivalent to in-person visits.

## Discussion

This pilot study confirmed the feasibility of telephone-based telemedicine in a public health setting. While reported satisfaction with telemedicine appeared high, similar to in-person visits, these findings are purely descriptive. The small sample size and imbalances preclude statistical inference, serving instead to generate hypotheses for future investigation. However, in-person visits showed greater agreement with negative statements, suggesting that logistical challenges ‒ such as longer waiting times, travel burdens, and emotional stress ‒ may negatively impact the patient experience.

These differences in patient profiles ‒ specifically higher education and lower clinical acuity in the telemedicine group ‒ likely influenced the satisfaction independent of the visit modality. Nevertheless, this observation is consistent with findings from previous research by Miziara et al. involving breast cancer patients, where telemedicine was associated with high satisfaction and perceived quality of care.^12^ These results reinforce the potential of telemedicine as an effective and patient-centered tool in oncology care when appropriately implemented.

Beyond logistics, gaps in perceived goal attainment and comprehension likely reflect context and selection rather than modality. In-person visits often involve travel, waiting, and difficult news, increasing anxiety and impairing information processing. By contrast, telemedicine was typically offered to clinically stable, digitally ready patients and occurred later in the care journey (median time since diagnosis 15 vs. 6 months; Table 2), favoring more focused conversations and higher perceived understanding.

Although patients reported ease of internet use, telemedicine still faces accessibility barriers, particularly concerning internet availability, device access, and digital literacy. Older adults and those in rural areas may lack the necessary connectivity or video-capable devices, limiting the feasibility of virtual visits.^4^^,^^16^ Additionally, the absence of physical contact remains a critical limitation, particularly in oncology, where clinical examinations play a key role in decision-making.^17^ Emotional aspects, such as breaking bad news or providing psychological support, were also perceived as more challenging in a virtual setting by providers, as reported by other studies.^17^ In the current study, the authors observed that the majority of patients possessed consistent internet access and characterized their usage as intuitive or highly accessible. This baseline digital readiness likely served as a key facilitator for the high levels of acceptability and patient satisfaction reported regarding the telemedicine intervention.

Despite these limitations, the descriptive data point to preliminary cost and convenience benefits for the telemedicine group. In this specific sample, in-person care was associated with reported out-of-pocket costs ‒ 7/9 (77.8%) reported expenses for transport, food, or other items versus 1/7 (14.3%) with telemedicine (Table 3). While these economic observations are based on self-reports from a small cohort and preclude generalizing cost-effectiveness conclusions, patients living far from the hospital or with mobility restrictions particularly benefited from avoiding these expenditures and the associated travel burden. This finding was similar in other studies.^12^^,^^18^ By eliminating the need for travel in routine visits, such as test result discussions, telemedicine optimized time efficiency for both patients and healthcare providers.^12^^,^^17^ Although the small sample size and potential confounders must be considered, these logistical benefits likely contributed to the high satisfaction observed with telemedicine.

The adapted PSQ-18 questionnaire, used to assess patient perceptions, required modifications to accommodate telemedicine visits. Originally developed for in-person care, its adaptation underscores the challenge of designing instruments that accurately evaluate both visit modalities. A potential solution would be to create hybrid questionnaires that include common questions while addressing the unique aspects of each format. Alternatively, validating adapted versions of existing instruments for telemedicine could ensure that key factors such as platform usability and communication quality are adequately assessed.^19^ Additionally, two cost-related questions were removed to better align with Brazil’s public healthcare system, improving the questionnaire’s applicability. Nevertheless, it is essential to note that employing an unvalidated, modified instrument constrains the reliability of these satisfaction metrics. Future research should focus on the psychometric validation of a telemedicine-specific instrument tailored to the SUS context. Furthermore, given the pilot nature of this work, item-level descriptive reporting was deemed appropriate, but formal validation remains a priority for future research.

Both patients and healthcare professionals expressed overall satisfaction with telemedicine, both through the survey and interviews, though some concerns persisted. Anxiety may influence patient perceptions, while privacy concerns and a perceived reduction in personal interaction could impact the patient-physician relationship.^20^ Some patients also reported difficulties in recognizing telemedicine calls, raising concerns about fraud and trust in virtual care. From the providers’ perspective, telemedicine was recognized as an effective tool for follow-ups and managing patients with mobility challenges. However, professionals emphasized its limitations for physical exams and delivering bad news, reinforcing the importance of in-person care in specific scenarios, as perceived in other studies.^10^^,^^11^^,^^17^

Integrating telemedicine as a complementary strategy to in-person care appears promising, particularly for follow-ups and non-urgent visits.^10^ Expanding technological training for patients and healthcare professionals and improving access to video calls could enhance its effectiveness.^3^ However, it is important to note that the present findings are specific to an audio-only model and may not fully extrapolate to video-based telemedicine. Unlike video, telephone consultations lack visual cues essential for assessing performance status (e.g., frailty, pallor) and non-verbal communication, which may affect both clinical safety margins and the depth of the patient-provider connection. Additionally, clear regulations on physician remuneration could promote its sustainability and long-term adoption.^21^ Future studies should explore broader quality-of-care metrics, including treatment adherence, surgical readmission rates, and patient retention, to further evaluate telemedicine’s impact.^10^^,^^22^

The absence of self-identified black participants in the telemedicine group raises concerns about disparities in access to virtual care and suggests possible digital exclusion based on racial and socioeconomic lines. This was accompanied by a higher education level in the telemedicine group, which may have influenced their ability to navigate digital platforms and engage with remote visits. Implementation should incorporate inclusion strategies (device/data support, assisted outreach, digital literacy initiatives, multimodal contact) and routine equity monitoring using standardized race/education categories. Without addressing these barriers, telemedicine may inadvertently widen disparities.

This study is primarily limited by its small sample size (n = 18) and non-random assignment, resulting in insufficient power for quantitative inference. The low recruitment rate (18/54) reflects real-world barriers to digital entry, stemming from both patient digital literacy gaps and institutional friction in adopting new telemedicine workflows. Consequently, the findings are descriptive and hypothesis-generating only, highlighting implementation feasibility rather than establishing comparative efficacy. This selection bias likely excluded socioeconomically vulnerable patients; a disparity underscored by the absence of self-identified black patients in the telemedicine cohort. Additionally, the imbalance in provider seniority ‒ with fellows conducting most telemedicine visits versus senior staff for in-person care ‒ introduces a significant confounder regarding patient satisfaction. Finally, the lack of a validated tool for this specific setting limits the generalizability of the satisfaction findings. A critical priority for future research is the development and formal psychometric validation of a telemedicine-specific satisfaction instrument tailored to the Brazilian public health context.

To our knowledge, this is the first pilot within Brazil’s public health system to evaluate the feasibility and patient satisfaction with telephone-based telemedicine for gynecologic oncology follow-up. The study reflects real-world audio-only practice and uses a mixed-methods design to integrate quantitative signals with qualitative context. The PSQ-18 was systematically translated and culturally adapted for the SUS context, with item-level reporting. Importantly, the study captured logistics outcomes of direct relevance to patients and services (e.g., travel-related expenses, missed work/school) and explicitly considered equity issues pertinent to digital inclusion.

## Conclusion

This study highlights telemedicine's feasibility and potential benefits as a complementary tool in gynecologic oncology care within Brazil’s public healthcare system. While it offers logistical advantages, reduces costs, and improves accessibility for patients with mobility limitations, its limitations must be acknowledged, particularly the inability to perform physical examinations and challenges in delivering sensitive information. Given the pilot scope and baseline imbalances, findings are preliminary and non-comparative. Future work should include an adequately powered randomized trial and the development, with proper validation, of a patient satisfaction instrument applicable to both in-person and telemedicine encounters.

## Institutional review board statement

This study was approved by the Institutional Research Ethics Committee of *Hospital Israelita Albert Einstein* under the Certificate of Presentation for Ethical Evaluation (CAAE 57,038,722.9.0000.0071).

## Informed consent statement

Informed consent was obtained from all subjects involved in the study. All participants provided written informed consent for the use of their clinical data for research purposes in accordance with the ethical standards of the institutional research committee and the Declaration of Helsinki.

## Declaration of generative AI and AI-assisted technologies in the writing process’

During the preparation of this work, the authors used ChatGPT in order to correct grammatical imprecisions and improve readability. After using this tool/service, the authors reviewed and edited the content as needed and take full responsibility for the content of the publication.

## Data availability

The datasets generated and analyzed during the current study are available from the corresponding author on reasonable request.

## Authors’ contributions

Conceptualization: R.F.H., F.M.P.; Methodology: R.F.H., F.M.P.; Validation: G.Y.C.; Formal Analysis: R.F.H., L.P.; Investigation: R.F.H.; Resources: N.A.; Data Curation: F.M.P.; Writing-Original Draft Preparation: R.F.H., F.M.P., G.Y.C.; Writing-Review & Editing: L.P., G.Y.C., J.L., F.P.P.L.L., E.C.; Supervision: R.M.M.; Project Administration: V.A.B.; All authors have read and agreed to the published version of the manuscript.

## Declaration of competing interest

The authors declare no conflicts of interest.

## Appendix Supplementary materials

Image, application 1

## Footnotes

Supplementary material associated with this article can be found, in the online version, at doi:10.1016/j.clinsp.2026.100968.
