# Supplementary material for: Feasibility of telephone-based telemedicine for gynecologic oncology follow-up in Brazil’s public health system: a descriptive pilot study
Source: Clinics (Sao Paulo). 2026 Apr 25;81:100968. doi: 10.1016/j.clinsp.2026.100968 (PMC13112396; doi:10.1016/j.clinsp.2026.100968)
Supplement: Supplementary file 1 [file mmc1.docx]

CLINICS-D-25-01303_Supplementary Material

**Supplemental Table 1** Process of translation of the PSQ.

| **Original PSQ-18** | **Adapted PSQ** | **Original PSQ-18** | **Final translation in Portuguese** | **Back-translation in English** | **Polarity** |
| --- | --- | --- | --- | --- | --- |
| 1 | 1 | Doctors are good about explaining the reason for medical tests | Médicos são bons explicando os motivos pelos exames | Doctors are good explaining the reasons behind tests | Positive |
| 2 | 2 | I think my doctor's office has everything needed to provide complete medical care | Eu acho que o consultório do meu médico tem tudo para me oferecer um atendimento médico completo | I believe my doctor's clinic has everything to offer a complete medical care | Positive |
| 3 | 3 | The medical care I have been receiving is just about perfect | O atendimento médico que recebo é praticamente perfeito | The medical care I receive is near-perfect | Positive |
| 4 | 4 | Sometimes doctors make me wonder if their diagnosis is correct | Às vezes questiono se o diagnóstico do meu médico está mesmo correto | Sometimes I question if my doctor's diagnosis is correct | Negative |
| 5^a^ | ‒ | I feel confident that I can get the medical care I need without being set back financially | Sinto que posso acessar o atendimento que preciso sem ser prejudicado financeiramente | I feel that I can access the care I need without being harmed financially | ‒ |
| 6 | 5 | When I go for medical care, they are careful to check everything when treating and examining me | Médicos são atenciosos e checam tudo que precisam para me tratar e examinar | Doctors are careful and check everything to treat and examine me | Positive |
| 7^a^ | ‒ | I have to pay for more of my medical care than I can afford | Eu preciso pagar mais pelo meu atendimento médico do que tenho condição | I need to pay more than I can afford for medical care | ‒ |
| 8 | 6 | I have easy access to the medical specialists I need | Tenho acesso fácil aos especialistas médicos que preciso | I have easy access to the medical specialists I need | Positive |
| 9 | 7 | Where I get medical care, people have to wait too long for emergency treatment | No serviço de emergência que uso, as pessoas demoram muito para serem atendidas | In the emergency service I use, it takes a lot of time for people to be seen | Negative |
| 10 | 8 | Doctors act too businesslike and impersonal toward me | Médicos são muito impessoais e falam complicado comigo | Doctors are very impersonal and formal with me | Negative |
| 11 | 9 | My doctors treat me in a very friendly and courteous manner | Médicos são muito amigáveis e atenciosos comigo | Doctors are very friendly and courteous with me | Positive |
| 12 | 10 | Those who provide my medical care sometimes hurry too much when they treat me | Às vezes, os profissionais de saúde têm pressa quando me atendem | Sometimes, health professionals are in a hurry when treating me | Negative |
| 13 | 11 | Doctors sometimes ignore what I tell them | Às vezes, médicos não ouvem o que eu falo) | Sometimes, doctors ignore what I say | Negative |
| 14 | 12 | I have some doubts about the ability of the doctors who treat me | Eu tenho algumas dúvidas sobre a competência dos médicos que me tratam | I have some doubts regarding my doctor's competence | Negative |
| 15 | 13 | Doctors usually spend plenty of time with me | Médicos geralmente passam bastante tempo comigo | Doctors generally spend a lot of time with me | Positive |
| 16 | 14 | I find it hard to get an appointment for medical care right away | Tenho dificuldade de conseguir uma consulta com meu médico rapidamente | I have some difficulties to get an appointment right away | Negative |
| 17 | 15 | I am dissatisfied with some things about the medical care I receive | Estou insatisfeito(a) com alguns aspectos do meu atendimento/tratamento | I am dissatisfied with some aspects of my care/treatment | Negative |
| 18 | 16 | I am able to get medical care whenever I need it | Consigo ser atendido(a) sempre que preciso | I can receive medical care whenever I need | Positive |

^a^ Items 5 and 7 were excluded from the final version.

**Supplemental Table 2** Interview guides.

|  | **Interview guide for patients** | **Interview guide for healthcare workers** |
| --- | --- | --- |
| 1 | What are your thoughts on telemedicine? | What are your thoughts on telemedicine? |
| 2 | In what situations do you find telemedicine useful? | In what situations do you find telemedicine useful? |
| 3 | When do you prefer telemedicine over an in-person visit? | In which patient scenarios or needs can telemedicine serve as a substitute for in-person visits? |
| 4 | How has telemedicine made things easier for you? | How has telemedicine made things easier for you? |
| 5 | Which telemedicine modality do you prefer? | Which telemedicine modality do you prefer for patient care? |
| 6 | What difficulties have you faced with telemedicine? | What do you dislike about telemedicine? |
| 7 | Based on your experience, how reliable is the technology? | Based on your experience, how reliable is the technology? |
| 8 | How easy is it for you to access telemedicine visits? | As a healthcare professional, how do you feel about prescribing medication via telemedicine? |
| 9 | Have you ever needed help joining a telemedicine visit? | Have you ever needed assistance setting up a video visit? |
| 10 | Do you have any difficulties with technology and/or the internet? | Do you find telemedicine easy or hard to use? |
| 11 | Do you think there are any barriers or challenges to using telemedicine? | In which situations have you offered telemedicine visits to a patient? |
| 12 | What did you find better about telemedicine compared to an in-person visit? | What challenges have you faced in telemedicine? |
| 13 | What did you find worse about telemedicine compared to an in-person visit? | What are the specific challenges of telemedicine in gynecologic oncology? |
| 14 | Has telemedicine helped you save money? | How do you feel about telemedicine visit fees? |

**Supplemental Table 3** COREQ Checklist.

|  | **Guide topic / question** | **Answer** |
| --- | --- | --- |
|  | **Domain 1: Research** |  |
| 1 | Interviewer/facilitator ‒ Who conducted the interviews? | Interviews were conducted by two members of the study team (one gynecologic oncology trainee and one advanced practice provider). |
| 2 | Credentials ‒ What were the researcher’s credentials? | Student doctor (final-year medical student) and MD, PhD, Gynecologic Oncologist. |
| 3 | Occupation ‒What was their occupation at the time? | Clinical staff in the gynecologic oncology service at a public hospital (SUS). |
| 4 | Gender ‒ Was the researcher male or female? | There were two researchers: one male and one female. |
| 5 | Experience and training ‒ What experience/training did the interviewer have? | The interviewer had prior training in patient interviewing, received a brief orientation on qualitative interviewing and the study guide, and conducted a pilot run with a mock interview. |
| 6 | Relationship established ‒ Was a relationship established prior to study commencement? | No prior relationship with participants beyond routine clinical contact; no prior relationship with interviewed providers outside the clinical team. |
| 7 | Participant knowledge of the interviewer ‒ What did participants know about the researcher? | Participants were informed that the interviewer was a member of the clinical team conducting a research interview to understand more about telemedicine services. |
| 8 | Interviewer characteristics ‒ Bias, assumptions, reasons for doing the research? | The team hypothesized that telephone telemedicine reduces the logistical burden of follow-up visits; we acknowledged a potential pro-telemedicine bias, which was addressed through the use of a structured interview guide and dual-coder analysis. |
|  | **Domain 2: Study design** |  |
| 9 | Methodological orientation and theory ‒ Which approach underpinned the study? | Thematic analysis (Braun & Clarke) within a pragmatic orientation; combined deductive (guide-derived) and inductive (emergent) codes. |
| 10 | Sampling ‒ How were participants selected? | Purposive sampling: all patients in the telemedicine arm were invited; purposive selection of clinicians (1 senior, 2 junior) to capture a range of experiences. |
| 11 | Method of approach ‒How were participants approached? | Patients: by phone shortly after their telephone visit or in person at clinic; clinicians: by phone or in-person invitation. |
| 12 | Sample size ‒ How many participants? | Three patients and three clinicians completed interviews. |
| 13 | Non-participation ‒ How many refused/dropped out? Why? | Three patients declined or could not be scheduled; and one clinician could not be reached, reasons included time constraints and lack of interest. |
| 14 | Setting of data collection ‒ Where were data collected? | Data was collected through telephone interviews. |
| 15 | Presence of non-participants ‒ Anyone else present? | No non-participants were present. |
| 16 | Description of sample ‒ Key characteristics? | Patient interviewees were female adults receiving gynecologic oncology follow-up via telephone; clinician interviewees included 1 advanced practice provider (> 10y) and 2 trainees (< 10y) in the same gynecologic oncology service. |
| 17 | Interview guide ‒ Were questions, prompts, pilot tests provided? | Structured/semi-structured guides adapted from Adake et al. were used; pilot-tested for clarity. The guides are provided in Supplement S2. |
| 18 | Repeat interviews ‒ Were repeat interviews carried out? | No repeat interviews were conducted. |
| 19 | Audio/visual recording ‒ Were the interviews recorded? | Interviews were audio-recorded with consent and transcribed verbatim. |
| 20 | Field notes ‒ Were field notes made? | Brief field notes were taken immediately post-interview to capture context and early impressions. |
| 21 | Duration ‒ What was the duration? | Median duration ~10 minutes. |
| 22 | Data saturation ‒ Was saturation discussed? | Saturation was not sought due to pilot scope and small N; findings are presented as hypothesis-generating. |
| 23 | Transcripts returned ‒ Were transcripts returned to participants for comment/correction? | No participant transcript checking was performed (pilot constraints, rapid cycle). |
|  | Domain 3: Analysis and findings |  |
| 24 | Number of data coders ‒ How many coders? | Two independent coders analyzed transcripts; discrepancies resolved by consensus. |
| 25 | Description of the coding tree ‒ Did you provide a coding tree? | A brief codebook (deductive + inductive codes) and theme map are provided in Supplement S2. |
| 26 | Derivation of themes ‒ Were themes identified in advance or derived from data? | Hybrid approach: deductive codes from the guide; additional themes inductively derived from the data. |
| 27 | Software ‒ What software was used? | Spreadsheets and collaborative memos. |
| 28 | Participant checking ‒ Did participants provide feedback on the findings? | No participant checking of findings (member checking) was performed (pilot constraints). |
| 29 | Quotations presented ‒ Were participant quotations used? | Yes; illustrative quotations are presented in Supplement S2, labeled by participant type (P1–P3; C1–C3). |
| 30 | Data and findings consistent ‒ Is there consistency between data and findings? | Themes are supported by multiple quotations across participants; divergences are noted where relevant. |
| 31 | Clarity of major themes ‒ Were major themes clearly presented? | Major themes: convenience/logistics, ease of use and tech support, limits of audio-only (non-verbal cues), access barriers/digital literacy. |
| 32 | Clarity of minor themes ‒ Were minor themes described? | Minor themes: call recognition/scam concerns, preference for video, telemedicine practical for mobility-limited patients, equity concerns. |

**Supplemental Table 4** Answers for the positive questions.

| **Question** | **In-person**  **(n = 10)** | **Phone**  **(n = 8)** |
| --- | --- | --- |
| 1. Doctors are good at explaining the reasons for medical tests |  |  |
| Strongly agree | 10 (100.0) | 8 (100.0) |
| Partially agree | 0 (0.0) | 0 (0.0) |
| Neutral | 0 (0.0) | 0 (0.0) |
| Partially disagree | 0 (0.0) | 0 (0.0) |
| Strongly disagree | 0 (0.0) | 0 (0.0) |
| 2. I believe my doctor’s office/video platform/phone has everything needed |  |  |
| Strongly agree | 8 (80.0) | 4 (50.0) |
| Partially agree | 1 (10.0) | 4 (50.0) |
| Neutral | 1 (10.0) | 0 (0.0) |
| Partially disagree | 0 (0.0) | 0 (0.0) |
| Strongly disagree | 0 (0.0) | 0 (0.0) |
| 3. The treatment I have received is very good |  |  |
| Strongly agree | 10 (100.0) | 8 (100.0) |
| Partially agree | 0 (0.0) | 0 (0.0) |
| Neutral | 0 (0.0) | 0 (0.0) |
| Partially disagree | 0 (0.0) | 0 (0.0) |
| Strongly disagree | 0 (0.0) | 0 (0.0) |
| 5. When I go to the doctor, they are thorough and check everything necessary to treat and examine me |  |  |
| Strongly agree | 10 (100.0) | 8 (100.0) |
| Partially agree | 0 (0.0) | 0 (0.0) |
| Neutral | 0 (0.0) | 0 (0.0) |
| Partially disagree | 0 (0.0) | 0 (0.0) |
| Strongly disagree | 0 (0.0) | 0 (0.0) |
| 6. I have easy access to the medical specialists I need |  |  |
| Strongly agree | 6 (60.0) | 7 (87.5) |
| Partially agree | 2 (20.0) | 1 (12.5) |
| Neutral | 2 (20.0) | 0 (0.0) |
| Partially disagree | 0 (0.0) | 0 (0.0) |
| Strongly disagree | 0 (0.0) | 0 (0.0) |
| 9. My doctors treat me in a friendly and respectful manner |  |  |
| Strongly agree | 10 (100.0) | 8 (100.0) |
| Partially agree | 0 (0.0) | 0 (0.0) |
| Neutral | 0 (0.0) | 0 (0.0) |
| Partially disagree | 0 (0.0) | 0 (0.0) |
| Strongly disagree | 0 (0.0) | 0 (0.0) |
| 13. Doctors usually spend enough time with me |  |  |
| Strongly agree | 6 (60.0) | 4 (50.0) |
| Partially agree | 4 (40.0) | 1 (12.5) |
| Neutral | 0 (0.0) | 2 (25.0) |
| Partially disagree | 0 (0.0) | 1 (12.5) |
| Strongly disagree | 0 (0.0) | 0 (0.0) |
| 16. I can get medical treatment whenever I need it |  |  |
| Strongly agree | 10 (100.0) | 7 (87.5) |
| Partially agree | 0 (0.0) | 1 (12.5) |
| Neutral | 0 (0.0) | 0 (0.0) |
| Partially disagree | 0 (0.0) | 0 (0.0) |
| Strongly disagree | 0 (0.0) | 0 (0.0) |

Data presented as n (%); Pilot descriptive study; no statistical tests were performed.

**Supplemental Table 5** Answers for the negative questions.

| **Question** | **In-person**  **(n = 10)** | **Phone**  **(n = 8)** |
| --- | --- | --- |
| 4. Sometimes doctors make me wonder if their diagnosis is correct |  |  |
| Strongly agree | 3 (30.0) | 3 (37.5) |
| Partially agree | 1 (10.0) | 2 (25.0) |
| Neutral | 0 (0.0) | 0 (0.0) |
| Partially disagree | 2 (20.0) | 0 (0.0) |
| Strongly disagree | 4 (40.0) | 3 (37.5) |
| 7. Where I get medical care, people have to wait too long for emergency treatment |  |  |
| Strongly agree | 0 (0.0) | 1 (12.5) |
| Partially agree | 2 (20.0) | 1 (12.5) |
| Neutral | 1 (10.0) | 0 (0.0) |
| Partially disagree | 1 (10.0) | 2 (25.0) |
| Strongly disagree | 6 (60.0) | 4 (50.0) |
| 8. Doctors act too businesslike and impersonal toward me |  |  |
| Strongly agree | 0 (0.0) | 0 (0.0) |
| Partially agree | 0 (0.0) | 0 (0.0) |
| Neutral | 0 (0.0) | 0 (0.0) |
| Partially disagree | 1 (10.0) | 1 (12.5) |
| Strongly disagree | 9 (90.0) | 7 (87.5) |
| 10. Those who provide my medical care sometimes hurry too much when they treat me |  |  |
| Strongly agree | 1 (10.0) | 0 (0.0) |
| Partially agree | 0 (0.0) | 1 (12.5) |
| Neutral | 0 (0.0) | 1 (12.5) |
| Partially disagree | 0 (0.0) | 1 (12.5) |
| Strongly disagree | 9 (90.0) | 5 (62.5) |
| 11. Doctors sometimes ignore what I tell them |  |  |
| Strongly agree | 0 (0.0) | 0 (0.0) |
| Partially agree | 0 (0.0) | 0 (0.0) |
| Neutral | 0 (0.0) | 0 (0.0) |
| Partially disagree | 0 (0.0) | 0 (0.0) |
| Strongly disagree | 10 (100.0) | 8 (100.0) |
| 12. I have some doubts about the ability of the doctors who treat me |  |  |
| Strongly agree | 1 (10.0) | 0 (0.0) |
| Partially agree | 0 (0.0) | 0 (0.0) |
| Neutral | 0 (0.0) | 0 (0.0) |
| Partially disagree | 1 (10.0) | 0 (0.0) |
| Strongly disagree | 8 (80.0) | 8 (100.0) |
| 15. I am dissatisfied with some things about the medical care I receive |  |  |
| Strongly agree | 3 (30.0) | 0 (0.0) |
| Partially agree | 0 (0.0) | 0 (0.0) |
| Neutral | 0 (0.0) | 0 (0.0) |
| Partially disagree | 0 (0.0) | 0 (0.0) |
| Strongly disagree | 7 (70.0) | 8 (100.0) |

Data presented as n (%); Pilot descriptive study; no statistical tests were performed.

**Supplemental Table 6** Patients' comments.

| **In-person group** | **Telemedicine group** |
| --- | --- |
| “The team was very attentive, everyone treated me really well. Everyone at the hospital, in general, has treated me very well”. | “The last visit was by phone. I was able to clarify my doubts. I was satisfied that I didn’t have to travel to the hospital”. |
| “I really liked it, the hospital is very good and well-equipped. I was very well received”. | “I am happy with the Doctor's team, everyone treats me with clarity, and that makes me feel protected and supported. I can only be grateful for everything. I thank God and this blessed team that He has prepared for me. May God bless everyone who took care of me. Gratitude to all”. |
| “Wonderful doctors!” | “The care was excelente”. |
| “Very satisfied”. | “Great”. |
| “The care was excellent; I have nothing to complain about. I'm just waiting for my kidney surgery to be scheduled”. | “Great service, very attentive team who explained things in a simple way that we could understand. They also showed concern for my well-being after surgery by following up via telemedicine. Excellent doctors!” |
| “Thank you to all the doctors. I am grateful to you for my treatment”. |  |
